# The Association Between Sickle Cell Anemia and Cognitive Dysfunction: A Systematic Review

**DOI:** 10.7759/cureus.69104

**Published:** 2024-09-10

**Authors:** Mohammed Alpakra, Nazim F Hamed, Zainab E Almakki, Esmaeel Al Bakrah

**Affiliations:** 1 Oncology and Hematology, Armed Forces Hospital Southern Region, Khamis Mushayt, SAU; 2 General Pediatrics, Security Force Hospital, Dammam, SAU; 3 Hematology, Maternity and Children Hospital (MCH), Dammam, SAU; 4 Microbiology, Armed Forces Hospital Southern Region, Asir, SAU

**Keywords:** cognition, cognitive dysfunction, cognitive outcomes, neurological complications, sickle cell anemia

## Abstract

A kind of hemoglobinopathy known as sickle cell anemia (SCA) is characterized by aberrant hemoglobin molecules. The most frequent neurological side effects linked to SCA include neurocognitive dysfunction, asymptomatic cerebral infarction, and ischemic stroke. This study aims to investigate the relationship between SCA and cognitive dysfunction. We systematically searched electronic databases like PubMed, MEDLINE, Science Direct, and Scopus. Two independent reviewers screened and extracted data from eligible studies. Eighteen studies, including 2,457 participants in total and nearly half of them 1,151 (46.8%) were males, were included in our data. The prevalence of cognitive dysfunction in the adult population ranged from 11.5% to 70%. Cognitive dysfunction among adults was significantly associated with poorer educational status, reduced family income, decreased kidney function, older age, stroke history, and vasculopathy. The prevalence of cognitive dysfunction in children ranged from 10.2% to 68.2%. The decline in cognitive function among adults was significantly associated with children over the age of four, abnormal transcranial Doppler and previous stroke, school absence, age beyond 13, and increased BMI. Cognitive function deficiencies are a defining feature of SCA that affects people of all ages. These findings suggest that if cognitive decline is not slowed down, or better still, stopped, medical interventions targeting a variety of sequelae in this population will be ineffective. Future analyses of this population's cognition should evaluate the environmental and other biological variables.

## Introduction and background

Sickle cell anemia (SCA) is a hereditary condition that alters red blood cell structure. The protein that carries oxygen in the blood, hemoglobin, is produced by a mutation in the hemoglobin gene. Red blood cells in people with SCA are not round but resemble crescent moons. Red blood cells with this aberrant shape may become lodged in tiny blood vessels, resulting in discomfort, organ damage, and other issues [1].

In recent years, researchers have begun to investigate the potential link between SCA and cognitive dysfunction. Cognitive dysfunction refers to difficulties with thinking, learning, and memory that can impact a person's ability to perform everyday tasks. This area of research is important because it could help to better understand the full range of complications associated with SCA, and potentially lead to new treatments to improve cognitive function in affected individuals [2].

Numerous investigations have discovered proof linking SCA to cognitive impairment. For instance, compared to children in good health, children with SCA scored lower on cognitive function tests, according to a study that was published in the journal Pediatrics. The scientists hypothesized that this might be caused by SCA's effects on the brain, which include decreased oxygen and blood flow [3].

According to other research, people with SCA have a higher chance of having a stroke, which can harm the brain and impair cognitive function. Stroke is really one of the most frequent neurological side effects of SCA, affecting up to 10% of individuals by the time they are 20 years old [4].

There exist multiple plausible pathways via which SCA may result in cognitive impairment. One theory is that the aberrant morphology of the red blood cells may restrict blood flow to the brain, which would reduce the amount of nutrients and oxygen that are delivered. This might lead to disruption of neuronal circuits and injury to brain cells, which would impair cognitive function [5].

Another potential mechanism is inflammation. Individuals with SCA have higher levels of inflammation in their bodies, which has been linked to cognitive dysfunction in other conditions such as Alzheimer's disease. Chronic inflammation can lead to oxidative stress and damage to brain cells, contributing to cognitive impairment [6].

Currently, there is no specific treatment for cognitive dysfunction in individuals with SCA. However, several strategies can help to manage the condition and improve cognitive function. These may include cognitive rehabilitation programs, educational support, and interventions to address other complications of SCA that could impact cognitive function such as stroke or pain [4].

In addition, ongoing research is exploring potential new treatments for cognitive dysfunction in SCA. For example, some studies have suggested that medications targeting inflammation or oxidative stress could help to protect the brain and improve cognitive function in affected individuals. Other research is focused on understanding the underlying mechanisms of cognitive dysfunction in SCA, which could lead to the development of more targeted therapies in the future [6].

Understanding the association between SCA and cognitive dysfunction has significant implications for both healthcare providers and individuals living with SCA. By clarifying this relationship, the study can contribute to the development of targeted interventions and support strategies to address cognitive impairment in these individuals. This can ultimately improve the quality of life and overall well-being of individuals with SCA, as cognitive impairment can impact daily functioning, educational attainment, and employment opportunities. Despite the well-documented effects of SCA on physical health, there is a lack of comprehensive understanding of its potential impact on cognitive function. This knowledge gap hinders the implementation of effective interventions and support systems for individuals with SCA who may be experiencing cognitive dysfunction. The aim of this study is to conduct a systematic review to consolidate existing evidence and determine the association between SCA and cognitive dysfunction. By synthesizing the available research, this study seeks to enhance our understanding of the relationship between SCA and cognitive impairment.

## Review

Methods

Search Strategy

Our systematic review was carried out in accordance with the Preferred Reporting Items for Systematic Reviews and Meta-Analyses (PRISMA) criteria in July 2024 [7]. We searched electronic databases such as MEDLINE, Scopus, Science Direct, and PubMed methodically. A comprehensive search method was developed to find papers on the connection between SCA and cognitive dysfunction. It combined Medical Subject Headings (MeSH) phrases with pertinent keywords. Examples of terms that could appear in the search are “Sickle cell anemia” OR “Sickle cell disease” alongside “Cognitive dysfunction” OR “cognition” OR “Neurocognitive dysfunction.” In order to capture a wider range of possible investigations, we also thought about incorporating sources from the grey literature, such as theses and conference proceedings.

Eligibility Criteria

Inclusion criteria: The studies eligible for inclusion must focus on the association between SCA and cognitive dysfunction. They should be published in English and involve human participants across all age groups, including both adults and pediatric populations. Additionally, the studies need to report specific cognitive outcomes or measures related to SCA and employ either observational or intervention study designs. These studies must provide clear and relevant data regarding the impact of SCA on cognitive function. Furthermore, the full-text articles of the studies must be accessible for review, and the research should have been conducted within the timeframe of 2019 to 2024.

Exclusion criteria: Conversely, studies were excluded if they do not directly explore the relationship between SCA and cognitive dysfunction or if they were not published in English. Research based on animal models or in vitro studies was not considered, nor were studies that lacked specific cognitive outcomes related to SCA. Also excluded are case reports, case series, reviews, commentaries, or editorials. Studies that focus on diseases or conditions unrelated to SCA and those that present insufficient or incomplete data on cognitive function in individuals with SCA were also discarded.

Data Extraction

It was necessary to verify the accuracy of the search results using Rayyan (QCRI) (Shaurya Polymers Private Limited, India) [8]. The titles and abstracts that were discovered during the search were evaluated for relevancy using the inclusion and exclusion criteria. Every article that met the inclusion requirements was thoroughly examined by the research team. Any issues were resolved by consensus. Important study information, including titles, authors, the release year, study setting, age, gender distribution, population type, tool used for cognitive dysfunction assessment, prevalence of cognitive dysfunction if mentioned, and main outcomes, were recorded using a predetermined data extraction form. A third-party assessment approach was developed to analyze the potential for bias.

Data Synthesis Strategy

A narrative synthesis approach was employed to amalgamate the results obtained from the research that was included. This involves grouping studies by research question and summarizing the key findings for each group. We also explored potential sources of variation across studies, such as differences in participant characteristics and cognition measurement methods.

Risk of Bias Assessment

The Joanna Briggs Institute (JBI) critical evaluation criteria for studies providing prevalence data were used to evaluate the study's quality [9]. This test consisted of nine questions. A good response received a score of 1, and a negative, unclear, or irrelevant response received a score of 0. Low, moderate, and high quality were assigned to scores that fall between 4 and 7, and 8 and above, accordingly. Disagreements were settled through conversation after researchers evaluated the studies' quality independently.

Results

Search Results

**Figure 1:**
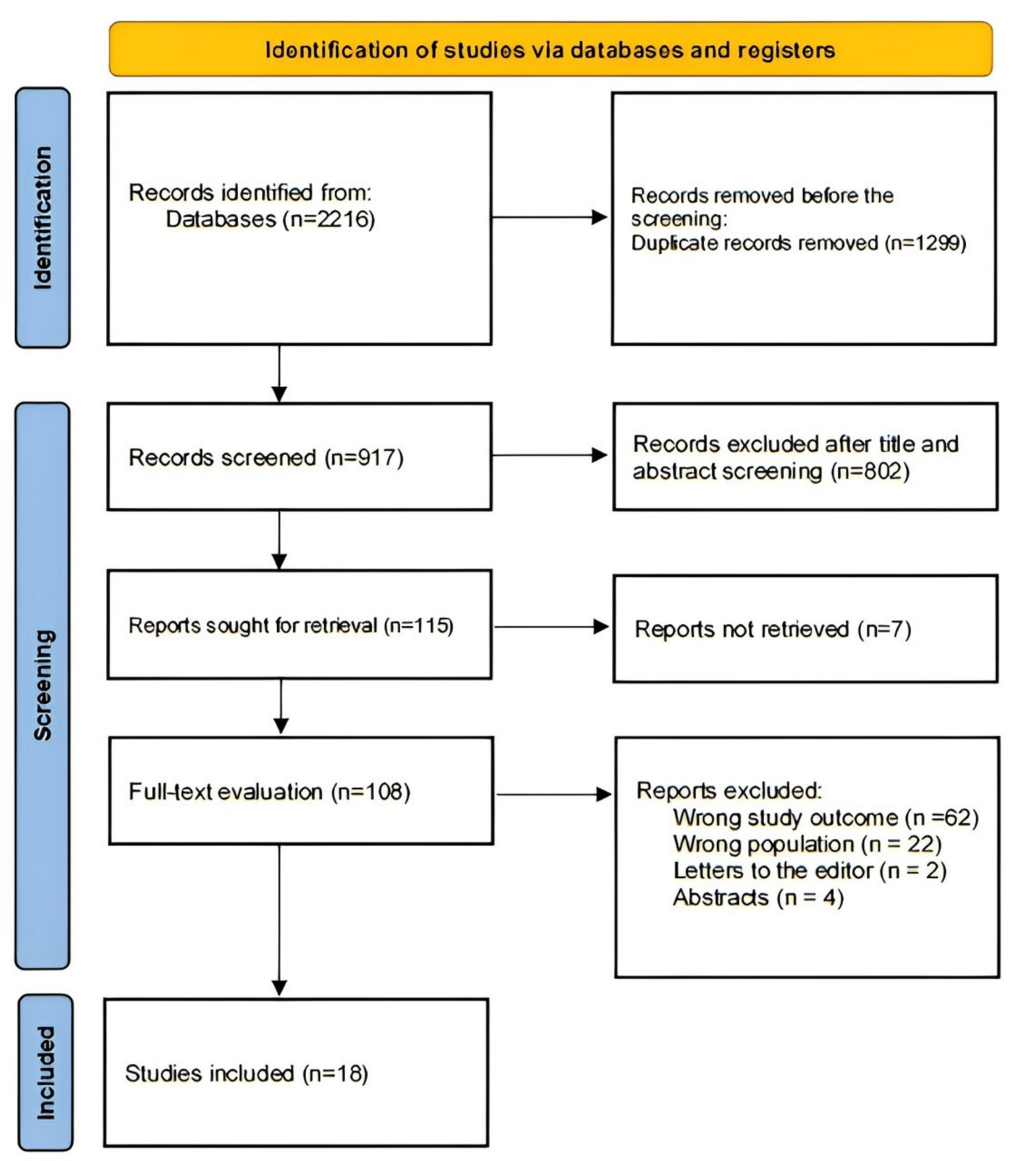
Study selection using a PRISMA diagram. PRISMA: Preferred Reporting Items for Systematic Reviews and Meta-Analyses

A thorough search turned up a total of 2,216 study papers after 1,299 duplicates were eliminated. Eight hundred two papers were deleted after the titles and abstracts of 917 studies were assessed. Of the 115 reports that needed to be retrieved, seven could not be found. After 108 papers were screened for full-text evaluation, 62 were rejected due to incorrect study results, 22 due to incorrect population type, two articles had editor's letters, and four contained abstracts. This systematic review's 18 research publications met the qualifying criterion. A summary of the process by which the research was selected is shown in Figure 1.

Sociodemographic Parameters of the Researched Subjects

Table 1 displays the sociodemographic information from the research articles. Our data includes eighteen trials with 2,457 individuals, 1,151 (46.8%) of whom were males [10-27]. Nine studies were cross-sectional studies [10,13,17,18,19,22,23,25,26], four were retrospective cohorts [14,16,20,21], three were case controls [11,12,27], and two were prospective cohorts [15,24]. Three studies were conducted in the USA [12,18,21], two in Brazil [10,27], two in Canada [13,15], two in Uganda [19,22], two in the UK [16,24], two in Tanzania [23,25], one in France [14], one in Turkey [17], one in Saudi Arabia [20], and one in Egypt [26].

**Table 1:** Sociodemographic characteristics of the involved population.

Study	Study design	Country	Participants	Mean age	Males (%)
Junqueira et al., 2024 [10]	Cross-sectional	Brazil	124	19-70	56 (45.2%)
Ampomah et al., 2022 [11]	Case-control	Ghana	63	18-50	29 (46%)
Chai et al., 2021 [12]	Case-control	USA	21	22.6 ± 8.9	8 (28%)
Forté et al., 2021 [13]	Cross-sectional	Canada	252	18-75	116 (46%)
Messimeris et al., 2024 [14]	Retrospective cohort	France	96	29.7 ± 10.2	42 (43.8%)
Couette et al., 2023 [15]	Prospective cohort	Canada	79	19-65	39 (49%)
Maduakor et al., 2021 [16]	Retrospective cohort	UK	303	38.8 ± 13.5	139 (46%)
Erdem et al., 2021 [17]	Cross-sectional	Turkey	100	16-32	40 (40%)
Partanen et al., 2020 [18]	Cross-sectional	USA	103	11 to 16	52 (50.5%)
Bangirana et al., 2024 [19]	Cross-sectional	Uganda	242	5.5 ± 2.9	124 (51.2%)
Basuni et al., 2024 [20]	Retrospective cohort	Saudi Arabia	27	<14	15 (55.6%)
Longoria et al., 2022 [21]	Retrospective cohort	USA	200	12.7 ± 3.56	109 (54.5%)
Green et al., 2019 [22]	Cross-sectional	Uganda	265	5.5 ± 2.9	139 (52.3%)
Matondo et al., 2020 [23]	Cross-sectional	Tanzania	313	9 to 15	112 (48%)
Clayden et al., 2023 [24]	Prospective cohort	UK	92	16.8 ± 6	49 (53.3%)
Jacob et al., 2022 [25]	Cross-sectional	Tanzania	73	11.9 ± 2.8	41 (56.2%)
Youssry et al., 2022 [26]	Cross-sectional	Egypt	40	6 to 20	14 (35%)
Castro & Viana, 2019 [27]	Case-control	Brazil	64	10.8	27 (42.2%)

Clinical Findings

The clinical features are shown in Table 2. Eight investigations on adults with SCA [10-17] found that the prevalence of cognitive impairment varied from 11.5% [13] to 70% [10]. Cognitive dysfunction among adults was significantly associated with poorer educational status [10,11], reduced family income [10], decreasing kidney function [13], older age [13], stroke history [14,15,17], and vasculopathy [16]. 

**Table 2:** Clinical characteristics and findings of the included studies. *NM=Not-mentioned RQCST=Revised Quick Cognitive Screening, TSA=Tract-specific analysis, RUDAS=The Rowland Universal Dementia Assessment Scale, WASI-II=Wechsler Abbreviated Scale of Intelligence-II, BRIEF-P=Behavioral Rating Inventory for Executive Function–Preschool version.

Study	Population type	Cognitive function assessment	Prevalence of cognitive dysfunction	Main outcomes	JBI
Junqueira et al., 2024 [10]	Adults	MoCA	87 (70%)	The significant frequency and effect of cognitive decline among adult SCA patients, as well as the apparent influence of sociocultural factors on cognitive function, such as poorer educational status, reduced family income, and the need to begin working earlier.	Moderate
Ampomah et al., 2022 [11]	Adults	RQCST	NM	Patients with SCA had lower cognitive performance at baseline. Furthermore, patients with SCA, especially those with lower educational levels, have been shown to have declining cognitive ability over time.	Moderate
Chai et al., 2021 [12]	Adults	TSA	NM	In SCA patients, neurocognitive performance showed slower processing speed and weaker reaction inhibition skills than in controls.	Moderate
Forté et al., 2021 [13]	Adults	RUDAS	29 (11.5%)	Cognitive dysfunction assessment with the RUDAS showed a high rate of probable dementia in adult SCA patients, which was linked to decreasing kidney function and age.	Moderate
Messimeris et al., 2024 [14]	Adults	MoCA	51 (53%)	Though underreported by the patients, cognitive dysfunction is common in young adult patients with probable neurological morbidity or stroke associated with SCA.	High
Couette et al., 2023 [15]	Adults	MoCA	NM	The most severe and widespread cognitive abnormalities as well as the lowest levels of educational attainment were linked to childhood strokes.	Moderate
Maduakor et al., 2021 [16]	Adults	NM	NM	Individuals with SCA and ischemic stroke had significantly higher rates of cognitive impairment (p < 0.0001). Similarly, cerebral vasculopathy and ischemic stroke were associated (r = 0.24, p = 0.03).	Moderate
Erdem et al., 2021 [17]	Adults	MoCA	NM	Cognitive dysfunction was the most frequent neurologic symptom reported by adult Turkish SCA patients. One of the patients had experienced their first ischemic stroke in maturity. Two people had severe neurologic symptoms as a result of an ischemic stroke.	Moderate
Partanen et al., 2020 [18]	Children	WASI-II	NM	Exposure to hydroxycarbamide has been linked to neurocognitive skills, and disease-modifying therapy may help with cognitive issues. Furthermore, impairment spans across neurocognitive domains, necessitating screening for deficiencies to identify patients who require additional examination or intervention.	Moderate
Bangirana et al., 2024 [19]	Children	BRIEF-P	NM	Neurocognitive assessment in children with SCA compared to non-SCA siblings revealed worse SCA-related achievement among children over the age of four.	Moderate
Basuni et al., 2024 [20]	Children	NM	NM	Many patients experience neurological consequences, which highlights the importance of early discovery and treatment. Even when their HbF levels are high, some patients still have neurological problems, indicating the need for additional therapies.	Moderate
Longoria et al., 2022 [21]	Children	WASI-II	NM	When it comes to children and adolescents with SCA receiving current disease-modifying medication, transcranial Doppler should not be regarded as a risk factor for poor neurocognitive results in the absence of overt stroke.	High
Green et al., 2019 [22]	Children	NM	27 (10.2%)	Abnormal transcranial Doppler and previous stroke were substantially correlated with neurocognitive impairment. Numerous aberrant findings, such as neurocognitive impairment, are highly prevalent in children with SCA brain illness.	High
Matondo et al., 2020 [23]	Children	NM	213 (68.2%)	School absence, age beyond 13, and BMI are all linked to neurocognitive decline.	Moderate
Clayden et al., 2023 [24]	Children	WASI-II	NM	By applying graph analysis to diffusion MRI in individuals with SCA, they discovered abnormalities in structural connectivity that mediate the relationship between blood oxygenation and cognitive functioning.	Moderate
Jacob et al., 2022 [25]	Children	WASI-II	NM	Children with SCA showed lower cognitive function on MRI or vasculopathy, whether or not they had SCI. Vasculopathy and SCI do not seem to affect cognitive function.	Moderate
Youssry et al., 2022 [26]	Children	Stanford Binet IQ test fourth edition	10 (25%)	Patients with SCA frequently experience cognitive impairment. It might be as easy as starting hydroxyurea therapy early on to protect these individuals' mental faculties. This treatment should also lower hemolysis and lactate dehydrogenase.	Moderate
Castro & Viana, 2019 [27]	Children	WASI-II	NM	Children with SCA have substantial cognitive impairment, which persists even after adjusting for socioeconomic background.	Moderate

Ten studies included the pediatric population with SCA [18-27] and the prevalence of cognitive dysfunction in children ranged from 10.2% [22] to 68.2% [23]. The decline in cognitive function among adults was significantly associated with children over the age of four [19], abnormal transcranial Doppler and previous stroke [21,22], school absence [23], age beyond 13 [23], and increased BMI [23].

Using graph analysis and diffusion MRI in individuals with SCA, a study found abnormalities in structural connectivity that mediate the relationship between measurements of blood oxygenation and cognitive functioning. Many children with SCA suffer neurological effects, emphasizing the significance of early detection and treatment [20]. Graph analysis of diffusion MRI in individuals with SCA revealed abnormalities in structural connectivity that mediate the relationship between measurements of blood oxygenation and cognitive functioning [18,26].

Discussion

One of the most common negative effects of SCA is deficits in cognitive performance. This is the first systematic review to comprehensively investigate the relationship between SCA and the incidence of cognitive dysfunction in both adult and pediatric populations. However, we found a lack of epidemiological data on the prevalence of cognitive dysfunction in SCA patients.

In this review, eight studies included the adult population with SCA [10-17] and the prevalence of cognitive dysfunction in the adult population ranged from 11.5% [13] to 70% [10]. Cognitive dysfunction among adults was significantly associated with poorer educational status [10,11], reduced family income [10], decreasing kidney function [13], older age [13], stroke history [14,15,17], and vasculopathy [16]. One of the most severe side effects of SCA is stroke, which affects up to 24% of patients by the age of 45 [28]. By the age of 18, silent cerebral infarctions (SCI) affect 39% of patients, and in adults, they affect 53% of patients [29-31]. According to estimates, 33% of adults have cognitive impairment [32]. These cognitive deficiencies affect social functioning and enjoyment of life, and they frequently result in unemployment [33].

This variant was previously assumed to be innocuous due to its clinical pattern. A recent study found that the SCA is a distinct risk factor for the occurrence and prevalence of chronic renal illness and albuminuria [34]. 

Furthermore, multiple studies have found that African Americans with SCA had a much-increased risk of cardiovascular abnormalities, including atrial fibrillation [35]. Chronic renal illness [36] and atrial fibrillation [37] have been linked to a significantly higher incidence of dementia and cognitive impairment. A recent study discovered that young African Americans were considerably more likely than the healthy controls to have a silent brain infarction on an MRI.

In the current review, ten studies included the pediatric population with SCA [18-27] and the prevalence of cognitive dysfunction in children ranged from 10.2% [22] to 68.2% [23]. The decline in cognitive function among adults was significantly associated with children over the age of four [19], abnormal transcranial Doppler and previous stroke [21,22], school absence [23], age beyond 13 [23], and increased BMI [23]. Prussien et al. reported that analyses revealed that compared to preschoolers, school-aged children had significantly more cognitive deficiencies [38]. Fortunately, primary stroke prevention efforts combining transcranial Doppler screening in combination with either hydroxyurea medication, blood transfusion intervention, or both in high-risk children are lowering stroke incidence rates in children with SCA residing in higher-income countries [39]. Unfortunately, no primary preventative treatment is available for children with SCI, which afflict up to 39% of school-aged children [40]. More research is needed to determine the impact of medical medications used to reduce the risk of infarctions and anemia on cognition.

In this review, a study found abnormalities in connectivity between structures that mediate relationships between blood oxygenation and cognitive functioning in patients with SCA utilizing graphing and diffusion MRI [24]. Numerous studies have found that independent of hemolysis rate, children with SCA have poorer autoregulation of cerebral blood flow than healthy children [41]. For this reason, it is believed that prolonged brain hypoxia causes the reduced cognitive function seen in SCA patients [42].

The study highlights the need for future research to encompass more diverse populations, allowing for a deeper understanding of how socio-economic, geographic, and genetic factors influence cognitive outcomes in individuals with SC). Longitudinal studies are particularly suggested, as they can provide insights into how cognitive function evolves over time, taking into account aging, disease progression, and the effectiveness of treatment interventions. Additionally, fostering a multidisciplinary approach by involving healthcare providers from various specialties, such as neurology, hematology, psychology, and social work, can create a holistic care model that addresses both physical and cognitive health aspects.

Standardization of cognitive assessment tools is essential for ensuring consistency across studies. Researchers are encouraged to focus on early interventions and preventative strategies tailored for children and adolescents with SCA, which could mitigate cognitive decline. It is also crucial to explore the underlying pathophysiological mechanisms that connect SCA and cognitive dysfunction through brain imaging studies and neuropsychological assessments. Furthermore, examining how different treatments-like hydroxyurea and blood transfusions-affect cognitive functions can provide valuable insights.

The implications of this research extend beyond the clinical realm; findings may inform health policies geared toward improving care quality for individuals with SCA, ensuring that cognitive health resources and support services are accessible. The discovery of a significant association between SCA and cognitive dysfunction could inspire the creation of targeted cognitive interventions aimed at enhancing the quality of life of affected individuals. In addition, integrating mental health services into the overall care plan may become necessary, recognizing the potential for cognitive challenges to accompany anxiety and depression.

Educational institutions might also benefit from this research by establishing support systems for students with SCA, thereby promoting inclusivity and academic success. Furthermore, the study may prompt increased funding and prioritization for cognitive dysfunction research in the SCA population, focusing on both prevention and treatment strategies. There may also be a need for enhanced training for healthcare providers regarding the cognitive implications associated with SCA, ensuring better patient management. Finally, raising public awareness about SCA and its cognitive implications can foster a greater understanding within the community, ultimately supporting individuals and families affected by this condition.

## Conclusions

The review's conclusions unequivocally demonstrate that cognitive function deficiencies are a defining characteristic of SCA, impacting individuals across all age groups. This pervasive cognitive impairment not only complicates the clinical picture of SCA but also profoundly affects the quality of life and functional independence of those diagnosed with the condition. The findings underscore a crucial implication: without effective strategies to slow or ideally halt cognitive decline, any medical interventions aimed at addressing various associated symptoms and complications of SCA are likely to be rendered ineffective. This highlights an urgent need for a multifaceted approach to treatment that prioritizes cognitive health alongside other medical concerns.

Moreover, the implications of these findings extend beyond immediate clinical concerns. They call into question the current standards of care and suggest the need for a reevaluation of therapeutic protocols that may not adequately address the cognitive dimensions of SCA. Upcoming research should not only seek to delineate the mechanisms underlying cognitive deficits in SCA but also explore how these deficiencies interact with other clinical features of the disease.
